# A phase I study of pegylated liposomal doxorubicin and temsirolimus in patients with refractory solid malignancies

**DOI:** 10.1007/s00280-014-2493-x

**Published:** 2014-06-11

**Authors:** Andrea Wang-Gillam, Nilay Thakkar, A. Craig Lockhart, Kerry Williams, Maria Baggstrom, Michael Naughton, Rama Suresh, Cynthia Ma, Benjamin Tan, Wooin Lee, Xuntian Jiang, Tibu Mwandoro, Lauren Trull, Stefanie Belanger, Allison N. Creekmore, Feng Gao, Paula M. Fracasso, Joel Picus

**Affiliations:** 1 Department of Medicine, Siteman Cancer Center, Washington University School of Medicine, 660 South Euclid Ave., Campus Box 8056, St. Louis, MO 63110 USA; 2 Department of Pharmaceutical Sciences, College of Pharmacy, University of Kentucky, Lexington, KY USA; 3 Banner Health Oncology Services, Northern Colorado, CO USA; 4 Diabetic and Cardiovascular Disease Center, Washington University School of Medicine, St. Louis, MO USA; 5 Siteman Cancer Center, Washington University School of Medicine, St. Louis, MO USA; 6 Division of Biostatistics, Washington University School of Medicine, St. Louis, MO USA; 7 Department of Medicine and the UVA Cancer Center, University of Virginia, Charlottesville, VA USA

**Keywords:** Pegylated liposomal doxorubicin, Temsirolimus, Phase I study, Refractory malignancies

## Abstract

This study aimed to determine the maximum-tolerated dose and dose-limiting toxicities of pegylated liposomal doxorubicin (PLD) in combination with temsirolimus (T) in patients with refractory solid tumors. Using a standard “3+3” dose escalation design, 23 patients were enrolled in three dosing cohorts in this phase I study. The starting dose level was PLD at 30 mg/m^2^ every 4 weeks and T at 20 mg weekly. Pharmacokinetics (PK) of doxorubicin were evaluated for patients in the expansion cohort. The most common treatment-related adverse events of all grades were mucositis/stomatitis (69.6 %), anorexia (52.2 %), thrombocytopenia (52.2 %), and fatigue (47.8 %). The recommended doses of this combination for phase II studies are 25 mg/m^2^ PLD and 25 mg T. PK analyses suggested increased exposure of doxorubicin in this combination regimen compared to doxorubicin administered as a single agent, possibly due to PK drug interactions. Out of 18 patients evaluable for a treatment response, two had partial responses (PR) (breast cancer and hepatocellular carcinoma) and six had stable disease (SD). Two patients remained on treatment for more than 1 year. The combination of PLD and T is tolerable, and the treatment resulted in clinical benefit. The combination regimen should be further explored in appropriate tumor types.

**Electronic supplementary material:**

The online version of this article (doi:10.1007/s00280-014-2493-x) contains supplementary material, which is available to authorized users.

## Introduction

The mammalian target of rapamycin (mTOR) acts downstream of the phosphatidyl-inositol 3 kinase (PI3K)/Akt pathway and plays a key role in the signaling of malignant cell proliferation, differentiation, migration, and survival [1]. Given that an overactive PI3K pathway is frequently implicated in the development and progression of a variety of malignancies, therapeutic targeting of crucial mediators along the PI3K signaling axis, such as mTOR, is an attractive strategy for cancer therapy.

Temsirolimus (T) (CCI-779, Torisel^®^ Pfizer Inc.) is a soluble ester analog of sirolimus and exerts its antitumor effect by selectively inhibiting mTOR. Temsirolimus received approval from the Food and Drug Administration (FDA) for treating patients with advanced renal cell carcinoma, based on a pivotal Phase 3 study demonstrating that patients receiving T had superior median overall survival when compared to those receiving interferon alpha alone or a combination of the two therapies (10.9, 7.3, and 8.4 months, respectively, *p* = 0.008) [2]. In addition, fewer patients in the T group experienced serious adverse events than the other two groups [2]. A second mTOR inhibitor everolimus (RAD-001, Afinitor^®^ Norvartis Pharmaceuticals Co.) has also been approved for advanced renal cell carcinoma after sunitinib or sorafenib treatment failure and for progressive neuroendocrine tumors of pancreatic origin. Therefore, mTOR inhibition is a proven antitumor strategy and is being evaluated in clinical trials for other cancer indications.

Doxorubicin is a cytotoxic anthracycline antibiotic isolated from *Streptomyces peucetius* var. *caesius*, and exerts its antitumor effect via inhibition of topoisomerase II [3]. In comparison with conventional or liposomal doxorubicin formulations, the pegylated liposomal doxorubicin (PLD) has extended blood circulation time, improved tumor localization, and better tolerance than conventional formulations [4]. Since its FDA approval, PLD is prescribed for refractory metastatic ovarian cancer and AIDS-related Kaposi Sarcoma [5, 6].

Several reports have indicated that mTOR inhibition may enhance the antitumor effects of cytotoxic agents in an additive or synergistic manner when tested in vitro and in vivo models of multiple types of human cancers [7, 8]. In particular, one preclinical study suggested that mTOR inhibition can reverse doxorubicin resistance conferred by PTEN mutation/Akt activation [9].

Based on the evidence suggesting potential synergy between mTOR inhibition and doxorubicin, we conducted a phase I clinical study evaluating the combination of PLD and T in patients with refractory solid malignancies. Here, we report the results on the safety, tolerability, pharmacokinetics (PK), and efficacy of this combination regimen.

## Methods

### Study design

This phase I study was an open-labeled, dose escalation study of PLD and T in patients with refractory tumors. The starting dose level (cohort 1) for the study was 30 mg/m^2^ PLD every 4 weeks and 20 mg T weekly (Table 1). A single treatment cycle was defined as 4 weeks, and dose-limiting toxicities (DLTs) were defined during the first cycle only. If none out of three patients in a cohort experienced DLT, then three patients proceeded to the next dose level. If two or more patients experienced DLT, then dose escalation was stopped. If one patient developed DLT, then three more patients entered the same dose level. The maximally tolerated dose (MTD) was defined as one dose level immediately below the dose level at which two patients of a cohort (of 2–6 patients) experienced DLT during the first cycle. Baseline imaging was performed within 28 days prior to starting the study and repeated after every two treatment cycles. Tumor response was assessed by the Response Evaluation Criteria in Solid Tumors (RECIST) 1.0 guidelines. All responses were confirmed by repeat imaging in 4 weeks. Toxicities were graded according to National Cancer institute Common Terminology Criteria for Adverse Events (NCI CTCAE), version 3.0. Hematological DLTs were defined as grade 4 neutropenia of ≥7 days duration, febrile neutropenia of any duration with temperature above 38.5 °C, grade 4 anemia, or grade 4 thrombocytopenia which required transfusion therapy on more than two occasions in 7 days. Non-hematological DLTs were defined as Grade 3 or 4 toxicity with the specific exceptions of nausea, vomiting, or anorexia. Grade 3 triglycerides were only considered a DLT after the administration of appropriate lipid-lowering agents. A delay of treatment for ≥14 days (consecutive or cumulative) prior to beginning cycle 2 was considered a DLT.

**Table 1:** Dose escalation schema

Dose levels	Pegylated liposomal doxorubicin (mg/m^2^) every 4 weeks	Temsirolimus (mg) weekly
1	30	20
1A	25	20
2A^#^	25	25

^**#**^Includes an expansion cohort

### Patient population

Patients were eligible to participate if they had a histological or cytological diagnosis of a malignant solid tumor that had progressed on standard therapies. Eligibility criteria included the following: life expectancy of at least 8 weeks; Eastern Cooperative Oncology Group (ECOG) performance status (PS) ≤2; age ≥18 years; measurable or evaluable disease as defined by RECIST; adequate bone marrow function [absolute neutrophil count (ANC) ≥1,500/mm^3^, hemoglobin ≥9 g/dL, and platelet count ≥100,000/mm^3^], adequate renal function (serum creatinine ≤2.0 mg/dL or creatinine clearance ≥60 ml/min/1.73^2^); adequate hepatic function [aspartate aminotransferase (AST), alanine aminotransferase (ALT) level, alkaline phosphatase ≤2.5× institutional upper limit of normal (ULN), and total bilirubin level ≤1.5 × ULN]; fasting cholesterol level ≤350 mg/dL; fasting triglyceride ≤400 mg/dL; albumin <3 mg/dL; and no active central nervous system (CNS) metastases, and normal left ventricular ejection fraction (LVEF ≥ 50 %) by multigated acquisition scan (MUGA). Patients may not have had chemotherapy or radiotherapy within 4 weeks of starting the protocol treatment. Patients must have provided signed and dated informed consent forms approved by the Washington University Human Research Protection Office (HRPO). All patients who received at least one treatment were included in the safety analysis. The study was conducted under the auspices of the Siteman Cancer Center Quality Assurance and Safety Monitoring Committee.

### Treatment administration and dose escalation procedure

Patients received PLD intravenously once every 4 weeks and T by intravenous infusion weekly. No treatment cycle was started until ANC ≥1,500/mm^3^, the platelets count ≥100,000/mm^3^, hemoglobin ≥9.0 g/dL, and all other treatment-related toxicities were grade ≤1. If, after the appropriate dose reductions, a treatment-related toxicity required a delay of ≥14 days before starting subsequent cycles, the patient was taken off study. If any grade ≥2 neutropenia or thrombocytopenia occurred at the start of a cycle, administration of both PLD and T was delayed (no more than 14 days) until the toxicity resolved to grade 1 or less. For patients experiencing toxicities, in subsequent cycles, PLD was given at a lower dose level while T could be resumed at the prior dose level or a reduced dose level at the discretion of the treating physician. For any occurrences of neutropenic fever, administration of either both drugs (at the start of a cycle) or T (during a cycle) was delayed until it resolved to grade ≤1 and then both drugs were reduced by one dose level. Once the dose was reduced for a subject, all subsequent cycles for PLD were administered at the reduced dose.

If grade ≥2 stomatitis/mucositis persisted at the start of a cycle, administration of both drugs was delayed until the toxicity resolved to grade ≤1, and the drug deemed responsible for the stomatitis/mucositis was reduced by one dose level in the current and subsequent cycles. If neither drug was identified as being responsible for the adverse events, then both drugs were reduced by one dose level. If grade ≥2 stomatitis/mucositis occurred during the weeks 2–4 of a cycle, T was omitted from the regimen until the stomatitis/mucositis resolved to grade ≤1; then, the drug deemed responsible for the adverse event was reduced in the current and subsequent cycles.

### Pharmacokinetic (PK) analyses

The PK of doxorubicin were assessed by analyzing the concentrations of doxorubicin in plasma samples collected at 0 (pre-dose), 0.5, 1.5, and 5 h after the start of drug infusion and approximately 48, 96, and 168 h post-infusion. Plasma concentrations of doxorubicin were analyzed using a liquid chromatography mass spectrometry analytical method (detailed description of bioanalytical methods provided in supplementary materials). The PK parameters were estimated using non-compartmental methods. The area under the concentration–time curve from time 0 to the last sampling point (AUC_last_) was calculated using the linear trapezoidal method. The remaining area under the curve from the last time point to infinity was calculated using the equation AUC_last−∞_ = *C*_last_/*k*_el_, where *C*_last_ is the last-measured plasma concentration and *k*_el_ is the slope of the concentration versus time plot during the log-linear terminal phase. Total clearance (CL) and elimination half-life (*t*_1/2_) were calculated as follows: CL = dose/AUC, *t*_1/2_ = 0.693/*k*_el_.

### Statistical methods

The primary outcome of this study was assessment of toxicity. The results are summarized by simple descriptive summary statistics. The PK variables for doxorubicin obtained using non-compartmental methods are summarized using mean ± standard deviations.

## Results

### Patient characteristics

From April 2008 to June 2011, a total of 23 patients were enrolled and treated with PLD and T. Demographic information for the study participants is shown in Table 2. The median age of patients was 60 years (range 34–80 years). The majority of the patients were Caucasian (21 of 23), and 14 (60.9 %) patients were male. The distribution of ECOG PS of 0 and 1 was 21.7 and 65.2 %, respectively. Twenty-one (91.3 %) patients had received at least one line of prior chemotherapy for their cancer. The most common cancer diagnoses were colorectal cancer (30.4 %) and breast cancer (17.4 %).

**Table 2:** Baseline clinical and demographic characteristics of enrolled patients

Characteristic	Patients (*n* = *23*)	
	No.	%
Gender		
Male	14	60.9
Female	9	39.1
Age (years)		
Median	60	
Range	34–80	
Ethnicity		
Caucasian	21	91.3
African Americans	2	8.7
ECOG performance status		
0	5	21.7
1	15	65.2
2	3	13.1
Number of previous chemotherapy regimens (median, range)	4 (0–11)	
Previous radiation		
No	7	30.4
Yes	16	69.6
Primary tumor type		
Colorectal	7	30.4
Breast	4	17.4
Non-small cell lung	3	13.1
Hepatocellular	2	8.7
Others^#^	7	30.4

^**#**^One patient each for prostate, gallbladder, esophageal, parotid gland, adrenal gland, squamous cell carcinoma of the skin, and malignant solitary fibrous tumor

### Safety and toxicity

The treatment toxicities experienced during the first cycle of treatment are summarized in Table 3. Six patients were treated in dose level 1 cohort (PLD 30 mg/m^2^ and T 20 mg); however, one patient never received treatment as she was determined to be ineligible after enrollment. Of the remaining five patients, no DLTs were observed (Table 4). Interim analysis was performed on this cohort. Although there were no DLTs at this dose level, the majority of patients were unable to complete two full cycles of treatment for evaluation of response, so the decision was made to reduce the starting dose level of PLD from 30–25 mg/m^2^.

**Table 3:** Summary of common drug-related adverse events occurring during the first cycle of treatment

Grade of adverse event	Dose level											
	1 (*n* = 5)				1A (*n* = 6)				2A (*n* = 12)			
	Grade				Grade				Grade			
	1	2	3	4	1	2	3	4	1	2	3	4
Mucositis/stomatitis		3			1	2			7	3		
Anorexia	2	2			2	1			2	3		
Platelets	1				4				6		1	
Fatigue	1					2			3	4	1	
Leukocytes		1				1			3	4		
Nausea	1	2			1				4	1		
Hemoglobin	2				1	1			3	1		
Lymphocyte		1			1	2			1	1	3	
Albumin	2				2				2			
AST (SGOT)					1	1	1^#^		2	1		
Pain: headache	2				2	1			1			
ALT (SGTP)					2	2			1			
Dyspnea					1			1^#^	1	1	1	
Heartburn/dyspepsia	1	1			1				2			
Rash/desquamation	2	1			1					1		
Glucose (serum)							2^#^			1	1	
Neutrophils			1						1	2		
Triglyceride (serum)					1				1	2		
Pruritus	3	1										

^#^Dose-limiting toxicity

**Table 4:** Summary of reasons that subjects discontinued study participation

Reasons for treatment cessation	1 (*n* = 5)	1A (*n* = 6)	2A (*n* = 12)	Total (*n* = 23)
Disease progression	1	5	9	15
Investigator/patient decision	1	0	1	2
Adverse event	1	1^#^	0	2
Intercurrent illness	1	0	1	2
No further dose reduction allowed on study	1	0	1	2

^#^Dose-limiting toxicity

Six patients were enrolled in the dose level 1A cohort (PLD 25 mg/m^2^ and T 20 mg), and one patient experienced the following DLTs [elevated AST (SGOT), hyperglycemia, and dyspnea]. Dose escalation proceeded as per the study protocol to dose level 2A. Six patients were enrolled in cohort 2A (PLD 25 mg/m^2^ and T 25 mg). One patient did not receive any treatment. Among five treated patients in this cohort, none experienced DLTs; therefore, this dose level was expanded. Seven patients were enrolled in the expansion cohort, for a total of 12 patients treated at this dose level. Adverse events in each cohort are summarized in Table 3. The common treatment-related adverse events during cycle 1 were: mucositis/stomatitis (16/23, 69.6 %), anorexia (12/23, 52.2 %), platelets (12/23, 52.2 %), and fatigue (11/23, 47.8 %).

The reasons patients discontinued treatment in this study are summarized in Table 4. Fifteen patients (65.2 %) were taken off the study due to disease progression. Two patients were removed from the study based on the investigator/patient’s decisions. One patient in cohort 2A developed recurrent mucositis, and the subject was taken off the study because no further dose reduction for T was permissible.

### Pharmacokinetics

**Fig. 1:**
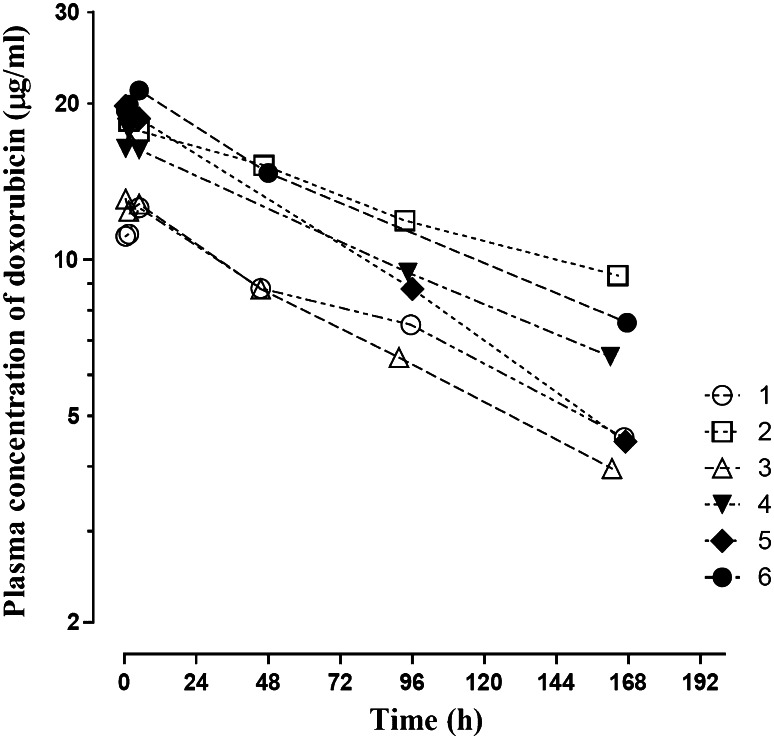
Individual plasma concentration versus time profiles of doxorubicin following intravenous administration of PLD (25 mg/m^2^) on the day 1 of cycle 2 in six patients

PK analyses of doxorubicin were performed following the intravenous infusion of PLD at 25 mg/m^2^ on day 1 of cycle 2 in 6 out of 7 patients in the expansion cohort of 2A (Fig. 1; Table 5). One patient in the expansion cohort did not have blood collected for PK on day 1 of cycle 2 due to disease progression. The plasma concentrations of doxorubicin were well above the lower quantitation limit at all time points. Although the PK blood sampling schedule was limited, it was possible to obtain reliable estimates of the terminal *t*_1/2_ from the regression analyses of the terminal log-linear decline (Fig. 1). Since the PK analyses were performed only on day 1 of cycle 2, it was not possible to assess the potential impact of the co-administered T on the PK of doxorubicin within our study. Thus, the results were compared to those available from the literature; in a previous study with the same 25 mg/m^2^ dose of PLD, the average terminal *t*_1/2_, AUC_0−∞,_ and CL values were 45.2 h (range 20.8–59.1), 609 µg_*_h/ml (range 227–887), and 80 ml/h (range 50–210), respectively [10]. The comparison of PK parameters obtained from our current study and similar studies in the literature is summarized in Table 5 [10, 11]. The results suggest that the systemic exposure of doxorubicin is much greater in patients receiving the combination therapy than in patients receiving PLD alone.

**Table 5:** Pharmacokinetic (PK) parameters of doxorubicin in six patients after intravenous administration of PLD (25 mg/m^2^) on day 1 of cycle 2

PK parameters	Median (ranges)	Median (ranges) [10, 11]
*t*_1/2_ (h)	115 (79–165)	45.2 (20.8–59.1)
AUC_0−∞_ (µg_*_h/mL)	2,600 (1,800–4,375)	609 (227–887)
CL (mL/h)	19 (11–26)	80 (50–210)

These patients also received weekly T (25 mg). The PK parameters from a previous study in patients receiving the 25 mg/m^2^ dose of PLD alone are also provided [10, 11]

AUC_0−∞_ = Area under the concentration versus time curve; *t*_1/2_ = Elimination half-life; CL = Total clearance

### Efficacy

**Fig. 2:**
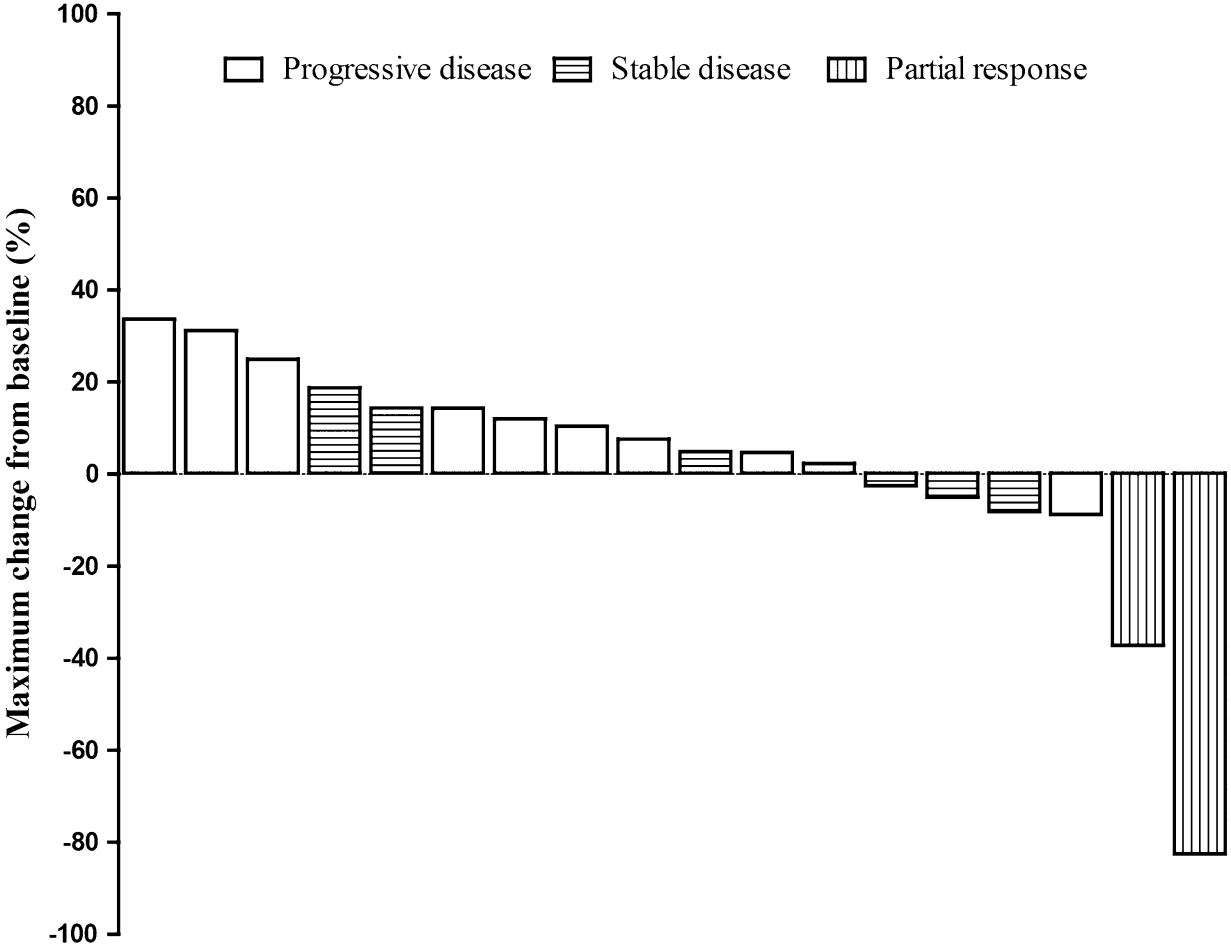
Waterfall plot of treatment response for 18 evaluable patients treated with PLD and T. Best tumor response (%) assessed by the Response Evaluation Criteria in Solid Tumors (RECIST) is plotted for individual patients. Several patients with less than 20 % increase in the maximum changes from the baseline were deemed to have progressive disease due to the appearance of one or more new lesions

Of 23 patients enrolled in the study, 18 were determined to be evaluable for treatment response because they had completed at least one cycle of treatment and one post-baseline imaging assessment. Five patients were not evaluable for treatment response due to the following reasons; no post-baseline imaging study (*n* = 2), consent withdrawal for grade 2 stomatitis (*n* = 1), discontinuation due to recurrent grade 2 stomatitis (*n* = 1), and discontinuation due to no further dose reduction available for PLD (*n* = 1). Figure 2 shows a waterfall plot of the treatment effect. Two (11.1 %) patients showed PR and six (33.3 %) patients displayed SD by RECIST. Several patients with less than 20 % increase in the maximum changes from the baseline were deemed to have progressive disease due to the appearance of one or more new lesions. One patient with a PR (dose cohort 2A) was on treatment for 6 months, and she was taken off study because there was no further dose reduction of T allowed for her recurrent stomatitis. This patient had breast cancer, and she had received three lines of chemotherapy before starting on this trial. The second patient with a PR (dose cohort 1) had hepatocellular cancer (HCC), and she was treated for 15 months and then removed for disease progression.

Among the seven patients with SD, the tumor types included HCC (*n* = 1), non-small cell lung cancer (*n* = 2), colorectal cancer (*n* = 2), squamous cell carcinoma of the skin (*n* = 1), and malignant solitary fibrous tumor (*n* = 1). The median treatment duration was 4 months (range 2–12 months). A patient with squamous cell carcinoma of the skin received a total of 12 cycles of treatment and had received 3 lines of chemotherapy prior to enrolling in the trial.

## Discussion

In this phase I study, we assessed the safety and tolerability of the combination of PLD and T in patients with refractory solid tumors. Based on our study, PLD at 25 mg/m^2^ every 4 weeks and T at 25 mg weekly appeared to be tolerable for patients with refractory solid tumors and is the recommended Phase II dose. The observed DLTs were elevated AST (SGOT), hyperglycemia, and dyspnea, and the most common adverse event of this combination drug regimen was mucositis/stomatitis, which was observed in 16 (69.6 %) patients but at grade ≤2 (Table 3).

A recently reported study independently determined that PLD at 30 mg/m^2^ every 4 weeks and T at 20 mg/m^2^ weekly as the MTD in patients with refractory or recurrent bone and soft tissue sarcoma [12]. The study enrolled 15 adult and pediatric patients, and their median age was 39 (range 9–70), much younger than those who participated in our current study (median 60, range 34–80, Table 2). In our study, the starting dose level was 30 mg/m^2^ PLD every 4 weeks and 20 mg T. However, the majority of patients who received this starting dose level could not complete two full cycles of treatment for a true evaluation of response, so PLD was reduced to 25 mg/m^2^ in the subsequent cohorts. Based on the recommended T single agent dose of 25 mg weekly, we further explored the combination of PLD at 25 mg/m^2^ and T at 25 mg weekly, which we determined to be the phase II dose for this combination regimen. Of note, our current study used the typical fixed mg dose of T approved by the FDA rather than mg/m^2^ used in the recent sarcoma study which probably required dose adjustment for the pediatric population enrolled [12]. Therefore, we believe that the phase II dose for the combination regimen from our study (PLD at 25 mg/m^2^ every 4 weeks and T at 25 mg weekly) may be more applicable to a general patient population with solid tumors.

The results from our PK analyses for doxorubicin also appear to be in line with the findings reported in the recent report in sarcoma patients [12]. Our results indicate that the weekly administration of T may have an impact on the PK profiles of doxorubicin when compared to the reported PK variables in the literature (Table 5). Our current study involved the PK assessment of doxorubicin only, due to the limited availability of blood samples from the enrolled patients. However, the recent report in sarcoma patients demonstrated that the concurrent administration of T and PLD resulted in increased systemic exposure (approximately twofold increase in AUC) of sirolimus, a major active metabolite of T [12]. These observed changes in the systemic exposure of doxorubicin and sirolimus are consistent with the previous reports that T, sirolimus, and doxorubicin are substrates for CYP3A4 and also for P-glycoprotein [13, 14]. Increased systemic exposure of doxorubicin and sirolimus when the two drugs were administered concurrently compared with single agent alone as well as some overlapping toxicities (e.g., mucositis) may have contributed to the adverse events observed in our study. Further investigations are warranted in order to probe the extent of the potential drug interactions for this combination regimen.

Emerging preclinical evidence suggests that mTOR inhibition can potentiate the antitumor efficacy of conventional cytotoxic agents in multiple tumor types in vivo and in vitro [7, 8]. Moreover, mTOR inhibition can overcome acquired cancer resistance to drugs targeting topoisomersase II [9, 15]. These preclinical observations may be clinically relevant as we observed clinical benefit in 44.4 % of patients; 6 stable diseases (SD, 33.3 %) and 2 partial responses (PR, 11.1 %) out of eighteen patients evaluable for treatment response. The duration of treatment for patients with SD was 4 months. Similarly, PLD and T have been combined with bevacizumab in another phase I study [16]. Out of 74 patients with advanced gynecologic and breast malignancies in that study, clinical benefit was achieved in 37.8 % of patients; 1 (1.4 %) complete response (CR), 14 (18.9 %) PR, and 13 (17.6 %) SD. Moreover, when assessed in patients with PI3K pathway aberrations (PI3K mutation or PTEN loss), this combination regimen resulted in clinical benefits (CR + PR + SD) in 52 % of patients; 9 (36 %) CR/PR and 4 (16 %) SD [16]. Our current study did not involve mutational analyses for PI3K pathway aberrations, but a subsequent study of this regimen enriched with patients selected for these molecular/genetic markers may be warranted.

In summary, the results from our current study demonstrated that the combination of PLD and T is tolerable and can yield clinical benefit. The combination regimen should be further explored in a phase II trial in patients with appropriate tumor types (e.g., advanced HCC, a disease with very limited therapeutic options). The efficacy of the combination regimen and the PK interaction between the two drugs warrant further exploration.

## Electronic supplementary material

Below is the link to the electronic supplementary material.

Supplementary material 1 (DOCX 12 kb)
