# Supplementary material for: A phase I study of pegylated liposomal doxorubicin and temsirolimus in patients with refractory solid malignancies
Source: Cancer Chemother Pharmacol. 2014 Jun 11;74(2):419–26. doi: 10.1007/s00280-014-2493-x (PMC4112045; doi:10.1007/s00280-014-2493-x)
Supplement: Supplementary file 1 — Supplementary material 1 (DOCX 12 kb) [file 280_2014_2493_MOESM1_ESM.docx]

**Supporting Information**

Bioanalytical methods

Liquid chromatography interfaced with tandem mass spectrometry (LC/MS/MS) assay for doxorubicin in human plasma was developed and validated according to FDA guidance and Good Laboratory Practice regulations. Samples were extracted using water and acetonitrile, 50 µg/ml of daunorubicin was used as internal standard and 5 µl of final extract was injected onto the HPLC column. The chromatography was performed at ambient temperature using a C18 guard column (4 x 3.0mm, Phenomenex) as a trapping column and XBridge C8 (3 x 50 mm, 3.5 µm particle size, Waters) as analytical column on a Shimadzu Prominence HPLC system. The mobile phases used to achieve analytical resolution were mobile phase A (0.1% formic acid in water) and mobile phase B (0.1% formic acid in methanol) at a flow rate of 0.6 ml/min. Mobile phase C (0.1% formic acid in acetonitrile/isopropanol (1:2)) was used to back-flush the trapping column after doxorubicin and daunorubicin were transferred to the analytical column. Doxorubicin and daunorubicin were monitored by an ABSciex 4000 QTRAP tandem mass spectrometer (MS/MS) equipped with an electrospray ion source in the positive ion mode and multiple-reaction monitoring (MRM) detection with precursor and product ion pair of m/z 544.3 and m/z 397.3 for doxorubicin; m/z 528.3 and m/z 363.3 for daunorubicin, respectively. The range of the assay for doxorubicin was 0.5 to 200 μg/mL. The intra-day assay accuracy ranged from −4.7% to 8.2%, with precision of ≤10.5% CV. The inter-day assay accuracy ranged from −0.9% to 4.5%, with precision of ≤7.4 % CV.
